# INCISIONAL HERNIA AFTER BARIATRIC SURGERY: ONLY THE PHYSICAL EXAMINATION IS ENOUGH?

**DOI:** 10.1590/0102-672020220002e1673

**Published:** 2022-09-09

**Authors:** Felipe Martin Bianco Rossi, Ricardo Moreno, Amarilys Luiza Druziani, Matheus Moreira Perez, Eduardo Possari, Renato Barretto Ferreira Da-Silva, Marçal Rossi

**Affiliations:** 1 RR Surgeons, Department of Surgery – Santo André (SP), Brazil; 2 Universidade São Caetano do Sul – São Caetano do Sul (SP), Brazil; 3 Centro Universitário Faculdade de Medicina do ABC, Faculty of Medicine of ABC – Santo André (SP), Brazil; 4 CDB Diagnostic Medicine – São Paulo (SP), Brazil.

**Keywords:** Incisional Hernia, Anastomosis, Roux-en-Y, Gastric Bypass, Ultrasonography, Hérnia Incisional, Anastomose em-Y de Roux, Derivação Gástrica, Ultrassonografia

## Abstract

**BACKGROUND:**

Incisional hernia is characterized by a bulging of the abdominal wall caused by the prolapse of intracavitary structures, such as a segment of the small intestine, through the trocar orifice. Ultrasonography and physical examination are used in the diagnosis of incisional hernia.

**AIMS:**

This study aimed to evaluate the difference between physical examination and abdominal ultrasonography at the diagnosis of incisional hernia in patients who underwent laparoscopic bariatric surgery.

**METHODS:**

A total of 123 patients who underwent Roux-en-Y gastric bypass type bariatric surgery performed by laparoscopy were analyzed for the presence or absence of hernia by physical and ultrasonography examination at each trocar incision site.

**RESULTS:**

In our results, a total of 7 hernias were detected by physical examination, while ultrasonography detected a total of 56 hernias in at least one of the incision sites. Lin's concordance analysis showed that the tests are not concordant. The association between body mass index and hernia detection (p=0.04 for physical examination and p=0.052 for ultrasonography) was observed. Ultrasonography detected more incisional hernias in 10-mm or larger trocars than in 5-mm trocars (p<0.0001, p<0.05). No differences were noted among the trocar types that were used.

**CONCLUSIONS:**

Abdominal ultrasonography showed to have a higher accuracy than physical examination, resulting in a substantial increase in incisional hernia detection at the trocar sites.

## RESUMO

**RACIONAL:**

A hérnia incisional é caracterizada por um abaulamento da parede abdominal causada por um prolapso das estruturas intracavitárias, como um segmento do intestino delgado, através de um orifício de trocarte. A ultrassonografia e o exame físico são usados no diagnóstico da hérnia incisional.

**OBJETIVOS:**

Avaliar a diferença entre o exame físico e a ultrassonografia abdominal no diagnóstico da hérnia incisional em pacientes submetidos a cirurgia bariátrica por videolaparoscopia.

**MÉTODOS:**

O total de 123 pacientes submetidos à cirurgia bariátrica, tipo derivação gástrica em Y de Roux, foram avaliados para a presença ou ausência de hérnia incisional por exame físico e ultrassonografia, nos sítios incisionais de cada trocarte.

**RESULTADOS:**

O total de sete hérnias foram detectados por exame físico, enquanto a ultrassonografia detectou um total de 56 hérnias em pelo menos um sítio incisional. A análise de concordância de Lin mostrou que os testes empregados não são concordantes. A associação entre o Índice de Massa Corpórea e a detecção de hérnia foi observada (p=0.04, para exame físico, p=0.052 para ultrassonografia). A ultrassonografia detectou mais hérnias incisionais em trocartes de 10 mm ou mais do que em trocartes de 5 mm (p<0,0001, p<0.05). Não foi observada diferença entre os tipos de trocartes empregados.

**CONCLUSÕES:**

A ultrassonografia abdominal demonstrou ter acurácia mais elevada que o exame físico, resultando em um aumento substancial na detecção de hérnia incisional nos locais de inserção dos trocartes.

## INTRODUCTION

Overweight and obesity are chronic comorbidities that occur worldwide, associated with several health effects, such as respiratory and cardiovascular diseases, diabetes, and diverse types of cancer. These conditions are characterized by the accumulation of visceral and subcutaneous adipose tissue and are defined by the body mass index (BMI; kg/m^2^)^24^. Over the past decade, high BMI is considered one of the principal health indicators worldwide, and the highest increase in relative incidence is one of the five risk factors most associated with death and decreased survival in the world population^11^.

The criteria established by the National Institute of Health for medical intervention suggest bariatric surgery for patients with a BMI >40 and patients with a BMI between 30 and 40 associated with comorbidities, such as type 2 diabetes^25,32^. Among the techniques of bariatric surgery, Roux-en-Y gastric bypass performed by laparoscopy is the most common. This technique became prevalent from the year 2000 onward, with more than 100,000 surgeries registered per year in the United States, according to the National Hospital Discharge Survey (1975–2005)^16^.

Although bariatric surgery is a safe technique, some postsurgical complications are associated with laparoscopic bariatric surgery. Incisional hernia (IH), also called trocar site hernia, is characterized by a bulging of the abdominal wall caused by the prolapse of intracavitary structures, such as a small intestine segment, through the orifice left by the instrument, resulting in organ incarceration and possible obstruction^8,22^. This condition results from a weakness or defect in the closure or healing of the musculoaponeurotic planes of the abdominal wall after a laparotomic incision, drainage orifices, or introduction of trocars. It requires correction surgery, resulting in increased hospitalization costs and new risks of complications^27^. Some risk factors associated with IH include BMI, the presence of infection at the incision site, thickness, type, depth, and site of the trocar^8^. Despite a sporadic incidence, resulting in approximately 1% of complications in laparoscopic surgery, some authors suggest an underestimation in these data as a result that most patients seek assistance only in symptomatic cases^21^.

Different approaches are used in the diagnosis of IH. Among them, computed tomography, ultrasonography, and physical examination are noteworthy. The European Hernia Society guidelines recommend for research purposes that the detection of IH be performed by imaging modalities, such as computed tomography and ultrasonography, to achieve more reliable results^15^. This recommendation differs from clinical practice, in which detection is performed predominantly by physical examination. Many studies use this more clinical approach, performing imaging diagnosis only in cases with inconclusive physical examination, while others consider only diagnoses confirmed by imaging^9^.

In addition, we hypothesized that different diagnostic approaches can influence the IH incidence rates meanly in the diagnosis of asymptomatic cases. Furthermore, diagnostic imaging techniques are believed to have a greater capacity to detect this complication when compared to physical examination alone.

The objective of this study was an active search for IH by physical examination and abdominal ultrasonography in patients who undergo bariatric surgery, to the diagnosis and incidence rates comparing both methods.

## METHODS

### Patients

This study included patients who were submitted to Roux-en-Y gastric bypass, with derivation in bariatric surgery performed by laparoscopy. All included patients signed the Informed Free Consent Form. The inclusion criteria for patients were as follows: BMI >35 with at least one associated comorbidity, or BMI ≥40, who was submitted to Roux-en-Y gastric bypass, performed by laparoscopy, with postoperatively follow-up for at least 6 months. Patients who experienced postoperative abdominoplasty and patients who had any complications in the postoperative period were excluded from the study. This study was approved by the Local Ethics Committee at Centro Universitáario FMABC, under registration n° 121/2018.

### Surgical Procedure

**Figure 1:**
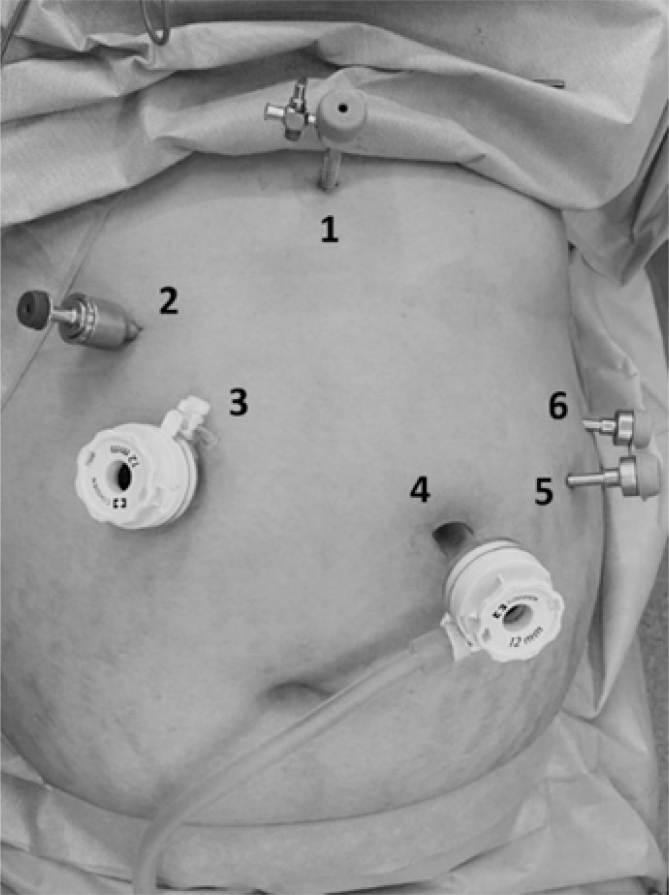
Anatomical sites of the trocar incisions.

Source: Own authorship.

Patients are placed in the supine position, slightly tilted head up. The surgeon and the camera are positioned to the patient's right and the assistant and the scrub nurse to the left; the video set is on the left above the patient's left upper limb. The access to the abdominal cavity is established with a reusable bladed trocar 10 mm long (Figure 1; No. 4) via the left supraumbilical region, about 20 cm below the xiphoid process and 2 cm from the midline, after puncture with a Veress needle and CO_2_ pneumoperitoneum at 15 mmHg. After cavity inventory, five additional trocars are introduced (Figure 1): a 5-mm trocar (No. 1) positioned just below the xiphoid process for inserting the liver retractor; a 5-mm trocar (No. 2) and a disposable 12-mm trocar (No. 3) placed 2 cm to the right of the patient's midline (close to the midclavicular line), which will be used by the main surgeon; a 5-mm trocar (No. 5) placed in the left midclavicular line at the level of the umbilical scar; and a 5-mm trocar (No. 6) placed in the left anterior axillary line.

### Trocar site closure

The aponeurotic closure was not performed.

### Types of trocar

We systematically employ permanent trocars in positions 1, 2, 4, 5, and 6. All the trocars used in position 4 are permanent and bladed. In position 3, the trocar used for stapling was a disposable, and it was employed two different types in this position: a bladed trocar (Medtronic^®^) and a bladeless, blunt-tip trocar (Ethicon Endosurgical Inc^®^).

### Evaluation of the abdominal wall for identification of incisional hernia at the trocar site

All patients were called up for an evaluation by the surgeon of the existence of IH in the trocar sites by physical examination and to answer a questionnaire about the presence or absence of hernia. After this evaluation, all patients were submitted to an abdominal wall ultrasonography, performed with the patient in the supine position and, if the presence or absence of a hernia was inconclusive, in the orthostatic position. This procedure was performed by a specialized radiologist.

### Statistical analyses

Qualitative variables were expressed in absolute and relative values. Quantitative variables were described using mean, minimum, and maximum values. The association between sample characteristics according to the presence of hernia before physical examination and the ultrasonography, and the difference of hernia incidence between 5 and 10 mm or 12-mm trocar was performed using Student's t-test. To assess the conformity between physical examination and ultrasonography for hernia detection, Lin's concordance, Pearson's correlation, and Bland-Altman's concordance correlation coefficient were employed. For all analyses, a 95% confidence level was used. The program used was Stata version 11.0.

## RESULTS

A total of 123 patients were included in the study. All patients were submitted to the inspection for the presence or absence of hernia by physical examination and ultrasonography at each incisional site caused by trocars in positions 1–6. The clinical characteristics and the examination data are described in Table 1.

**Table 1:** Patient's characteristics and hernia incidence.

Characteristics		Mean	Min–Max
Age (years)		39.23	21.00–62.00
Height (m)		1.62	1.47–1.85
Time since surgery (months)		13.83	1.50–75.00
Weight before surgery (kg)		108.82	80.0–147.00
Weight (kg)		69.48	49.00–97.00
Weight loss (kg)		39.34	8.00–66.10
Weight loss (%)		35.87	9.09–50.37
BMI (kg/m^2^)		26.40	20.20–36.50
Hernia presence with physical examination		N	%
Trocar 1 site			
	No	122	99.19
	Yes	1	0.81
Trocar 2 site			
	No	122	99.19
	Yes	1	0.81
Trocar 3 site			
	No	119	96.75
	Yes	4	3.25
Trocar 4 site			
	No	122	99.19
	Yes	1	0.81
Trocar 5 site			
	No	123	100
Trocar 6 site			
	No	123	100
Ultrasonography			
Trocar 1 site			
	No	121	98.37
	Yes	2	1.63
Trocar 2 site			
	No	121	99.18
	Yes	1	0.82
Trocar 3 site			
	No	107	86.99
	Yes	16	13.01
Trocar 4 site			
	No	88	71.54
	Yes	35	28.46
Trocar 5 site			
	No	121	98.37
	Yes	2	1.63
Trocar 6 site			
	No	123	100

BMI: body mass index.

Table 2 describes the association between patient characteristics and verification by physical examination and ultrasonography of the absence or presence of a hernia in at least one of the incision sites.

**Table 2:** Association between patients’ characteristics and presence or absence of hernia for physical examination and ultrasonography.

Characteristics	Hernia presence	Hernia absence	p-value*
Physical examination	Mean (95%CI)		
Age (years)	38.82 (36.85; 40.78)	46.00 (42.50; 49.50)	0.08
Height (m)	1.62 (1.61; 1.62)	1.60 (1.54; 1.67)	0.51
Control surgery	13.69 (12.14; 15.25)	16 (10.32; 21.68)	0.48
Weight before surgery	109.27 (106.44; 112.10)	101.43 (91.61; 111.24)	0.18
Weight	69.92 (67.97; 71.87)	62.24 (54.85; 69.63)	0.06
Weight loss (kg)	39.35 (37.33; 41.38)	39.19 (31.45; 46.93)	0.96
Weight loss (%)	35.71 (34.34; 37.08)	38.48 (32.66; 44.31)	0.33
BMI	26.54 (25.95; 27.12)	24.10 (22.79; 25.41)	0.04
**Ultrasonography**	**Median (95%CI)**		
Age (years)	38.88 (36.48; 41.28)	39.77 (36.62; 42.92)	0.65
Height (m)	1.62 (1.61; 1.64)	1.62 (1.60; 1.65)	0.87
Control surgery	14.05 (11.82; 16.27)	13.48 (11.82; 15.14)	0.71
Weight before surgery	108.82 (105.28; 112.36)	108.83 (104.40; 113.25)	0.10
Weight	70.37 (67.87; 72.88)	68.08 (65.16; 71.01)	0.24
Weight loss (kg)	38.45 (35.77; 41.12)	40.74 (38.01; 43.48)	0.25
Weight loss (%)	34.98 (33.12; 36.85)	37.25 (35.52; 38.98)	0.09
BMI	26.84 (26.04; 27.64)	25.71 (24.99; 26.43)	0.05

* Student's t-test; BMI: body mass index; CI: confidence interval.

To verify the conformity between physical examination and ultrasonography in the detection of IH, Lin's concordance, Pearson's correlation, and Bland-Altman's concordance correlation coefficient tests were performed. Results are shown in Table 3.

**Table 3:** Lin's concordance, Pearson's correlation, and Bland-Altman's concordance correlation coefficient between physical examination and ultrasonography in the detection of incisional hernia.

Method	Hernia presence		
	(95%CI)*	Pearson	Bland-Altman (95%CI)
Physical examination Ultrasonography	0.051 (-0.049; 0.151)	0.091	-0.333 (-1.35; 0.69)

* Lin's concordance coefficient; CI: confidence interval.

A total of seven hernias were detected by physical examination, while ultrasonography detected a total of 56 hernias in at least one of the incision sites. The trocar No. 4 (10 mm) puncture was the one that showed the greatest difference, having been detected by a physical examination in only one of the patients (0.81%), against 35 (28.46%) patients using ultrasonography technique. These results suggest a greater accuracy of this technique, confirmed by Lin's concordance analysis which indicated that the results in both procedures were not concordant with each other. An association was observed between BMI and hernia detection (p=0.04 for physical examination and p=0.052 for ultrasonography), suggesting that this characteristic may interfere with the accuracy of both methodologies.

The relationship between IH incidence and the size of the trocar used in the procedures was analyzed. Our results demonstrated an increase in IH detected by ultrasonography in the trocars with at least 10-mm compared to the 5-mm trocars. The physical examination analysis detected two (0.40%) and five (2.03%) IH, respectively, in 5- and 10-mm or larger trocars (p=0.25), two and were identified, by physical examination and ultrasonography. In contrast, the ultrasonography detected 5 (1.01%) and 51 (20.73%) IH, respectively, in 5- and 10-mm or larger trocars (p<0.0001).

Regarding the analysis of the two types of 12-mm trocars used in position 3, and the relation with the incidence of IH, a total of 88 (71.45%) patients were submitted to the surgical procedure using the bladed trocar. Among them, 3 (3.4%) presented IH diagnosed by physical examination and 11 (12.5%) had IH detected by ultrasonography. In contrast, 35 (28.45%) patients had the surgical procedure executed using the bladeless, blunt-tip trocar. In this group, we observed 1 (2.85%) case of IH detected by physical examination, and 5 (14.28%) patients presented IH when observed using ultrasonography. No significant difference in the incidence of IH was observed in the postoperative period when comparing both bladed and bladeless trocars.

The relationship between weight loss and incidence of IH was analyzed in the study. The patients were divided into two groups with a similar number of subjects. The first group was composed of patients who presented the lowest percent values of weight loss varying from 9.09 to 35.51% (62 subjects — weight loss mean of 30.02%). The second group was formed by patients with the higher percent values of weight loss varying from 35.54 to 50.37% (61 patients — weight loss mean of 41.62%). On the first group were identified two IH in the physical examination, contrasting with the ultrasonography that detected 24 IH in the patients from this group. In contrast, in the second group composed of the higher weight loss subjects, we observed 5 IH through physical examination and 32 IH using ultrasonography.

## DISCUSSION

There is some discordance in the literature regarding the incidence of IH. After bariatric surgery, most authors report an incidence between 0.2 and 1%. Schauer et al. presented two cases (0.7%) in 275 patients analyzed, with 1 patient requiring laparoscopic correction^28^. Chevalier et al. demonstrated that 4 (0.4%) out of 1000 patients operated had IH as late complications^3^. Dresel and co-authors presented a case of incisional trocar herniation in his first 100 (1%) cases^6^. Our study shows that the chosen diagnostic approach can influence these incidence rates, as a considerable number of patients can be asymptomatic and the IH not detected during physical examination — this has raised the question about the commonly clinical approach to adopt by the medical community to diagnosis and evaluate the presence of IH in patients after bariatric surgery.

The literature review showed that the actual incidence of incisional trocar hernia has been poorly studied through imaging examinations. Using exclusively physical examination can influence the diagnosis accuracy. Some factors are suggested to compromise the accuracy of this clinical approach as the thickness of the fatty panicle and the excess skin after weight loss in the postoperative period can lead to difficulty in the detection using palpation, especially in asymptomatic cases. In addition, the failure and delay in diagnosis, the patient's tolerance to asymptomatic hernia, and loss of follow-up also lead to under-diagnosis^20,30^. Bloemen et al. observed that, although neither physical examination nor ultrasonography had 100% sensitivity and specificity, the combination of both modalities increases the chance of detecting IH. In their findings, there was concordance between the two techniques because, individually, three false-negative rates were approximately 25% in both. Of a total of 456 examined patients, 82 (18%) had a hernia detected by physical examination, and 83 (18.2%) were detected by ultrasonography. Of these, 62 were detected using both techniques. For 20 patients, hernias were detected only by physical examination and 21 only by ultrasonography.

The authors pointed out that obesity could have influenced the false-negative rates^1^. In our results, we investigated the incidence of hernia by both methods at each incision site. A significant increase in the detection of IH was found when using the ultrasonography approach. In general, image methodologies to diagnose IH have been shown to be more precise than only physical examination — although some factors can influence the accuracy of physical examination, as discussed previously, and may interfere in the screening of IH in asymptomatic patients.

Ultrasonography is considered one of the cheapest and most accessible approaches among imaging techniques. Although the use of image methodologies to diagnose IH in some communities is limited and most clinicians are focused on the detection of symptomatic patients who may require treatment. An additional point to the selection of diagnostic procedure debates the divergence in the aims between clinicians (identifying symptomatic patients) and researchers (identifying all cases of IH) that may influence the choice of procedure adopted^21^. In this context, asymptomatic patients are notable cases, as even small hernias can dispose a greater risk of strangulation once incarcerated and when not early diagnosed may increase size over time and represent important implications for the patient's health^14^.

In addition, few studies have evaluated alternatives for closing the aponeurosis at the trocar sites in laparoscopic bariatric surgery. Chiu et al. retrospectively reviewed 752 patients (610 mini-bypass and 142 gastric bands). None of these patients had the aponeurosis of the trocar puncture closed. Instead, a plug using Surgicel^®^ was inserted into the muscle layer of the 10 and 12 mm diameter trocar incisions. Only two male patients in the gastric mini-bypass group developed IH (0.33%). In these two patients, the hernia occurred in the 12-mm trocar in the left midclavicular line, 2–3 cm below the costal margin, outside the left abdominal rectus muscle. These two patients did not develop intestinal obstruction and did not undergo hernia repair surgery^5^. Liu and McFadden demonstrated that no bleeding or hernia occurred in any of the five incisions in a series of seven obese patients who underwent laparoscopic Roux-en-Y gastric bypass using blunt-tipped trocars and without closure of the incision. This suggests that blunt trocars and nonclosure of the incisions are effective for obese patients^18^.

The type of trocar was also studied as a variant in the incidence of hernia. Controversially, the literature has shown different results. Chiong et al. observed the incidence of hernias after the use of dilatation trocars (Excel^®^ and VersaStep^®^). Out of 1,055 patients, 7 (0.66%) had a trocar herniation after an average follow-up period of 13 months^4^. Schmedt et al., in a prospective cohort study, compared the use of conical-tipped trocars with pyramid-tipped trocars. The prevalence of hernia was significantly reduced with the use of conical trocars (0.02% for conical trocars vs. 1.2% for pyramidal trocars)^29^. Johnson et al. studied the use of the VersaStep^®^ trocar (Covidien^®^) in 747 patients who underwent bariatric surgery and evaluated the incidence of hernia when the puncture was not closed. This trocar does not have a cutting function, but of opening the aponeurosis. The analysis did not detect hernias, although the study did not present which approach was used to evaluate the presence of hernias in the postoperative period^12^.

A previous literature review highlighted that using blunt trocars caused a decrease in the incidence rates than studies using bladed trocars^10^. However, in our findings, no significant differences were found in the comparison between bladed and blunt trocars utilized — it was detected cases of IH in both trocar types used among the patients studied. Supporting those finds, previous reports in the literature pointed to the incidence of IH even using a blunt-tip trocar^14,17^. Even though nonbladed trocars are considered less traumatic to the abdominal wall, other factors can influence the incidence of IH that may be related to the size of the trocar.

Considering that, some authors suggested a marked relationship between the size of the trocar and the incidence of hernia. Although the following data were related to gynecological surgery, in a study carried out by the American Association of Laparoscopic Gynecology, in 840 trocar hernias, about 725 (86.3%) occurred in places where the diameter was at least 10 mm. Only 92 (10.9%) hernias occurred in a puncture site that was between 8 and 10 mm in diameter. Only 23 (2.7%) cases were diagnosed in portals smaller than 8 mm^19^. Kadar et al., in another gynecological laparoscopy study, demonstrated that closing the aponeurosis in a 12-mm trocar site significantly reduced the rate of hernia development compared to when it was not closed^13^.

In our study, we observed a significant increase in IH cases in the sites that applied 10-mm or larger trocars when compared to 5-mm trocars using the ultrasonography. Other studies in the literature related the same findings as the present study — demonstrating that the size of trocars may influence the incidence of IH^13^.

The location of the trocars has also been studied and indicated as an important factor in the incidence of hernias. Plaus demonstrated that puncture sites outside the midline may be less susceptible to hernia due to overlapping muscle layers and aponeurosis^26^. Duron et al. reported that the lateral wall is composed of two fascial and muscular planes, making it theoretically less prone to dehiscence^7^.

The correlation between the incidence of IH and weight loss after bariatric surgery is poorly studied in the literature. Some authors discuss that the previous weight loss before the surgical procedures for the correction of IH may be beneficial^2,23^. In addition, there are not enough studies that investigated the effects of weight loss and the frequency of IH in the postoperative. Our study showed that in higher weight loss cases, there was a higher incidence of IH in the patients — in both protocols used for the diagnosis. This finding may be related to abdominal wall structure alterations due to higher body mass loss. In contrast, another hypothesis is the difficulty to diagnosis IH in patients who lost less weight after the surgical procedure. To better understand this correlation and the factors involved, further investigations are necessary.

There is no consensus about the actual incidence of IH or even established more effective guidelines to the diagnosis that includes the asymptomatic patients as a common clinical practice. In the literature, most studies only evaluated the incidence and reports of incisional trocar hernias in laparoscopic surgery and also in bariatric surgery. Similarly, just a few studies investigated the relationship between trocar hernia and the clinical characteristics of patients, such as chronic diseases, BMI, thickness, and type of trocar used. In addition, studies that demonstrated techniques for closing the hernia defect are even more scarce, especially when related to bariatric surgery^31^. Considering that, studies as the present one, exhibits an important role in the understanding of the actual incidence rates of IH as well as the factors that may influence during the surgery procedure.

## CONCLUSION

This is the first study in Brazil to report the differences in the incidence of IH according to the clinical procedure used for the diagnosis. The diagnosis of IH remains challenging, as there is no standard procedure that is effective to detect symptomatic and asymptomatic cases. According to our results, ultrasonography proved to be more accurate than physical examination, resulting in a substantial increase in hernia detection, thus suggesting the use of this approach in the clinical practice during the follow-up after laparoscopic bariatric surgeries. In the literature, there is a lack of consense in the actual number of IH prevalence, which is influenced by several factors including the diagnostic procedure adopted in the clinical practice. Therefore, it is important to highlight that there is an increased concern about the impact of IH cases in the public health system and the improvement in the management of the cases. Thus, our results may contribute to the understanding of the factors that influence the IH evolution after bariatric surgery and also the underestimation of the IH cases influenced by the clinical procedure.
